# Temporal patterns of genetic diversity in Kirtland’s warblers (*Dendroica kirtlandii*), the rarest songbird in North America

**DOI:** 10.1186/1472-6785-12-8

**Published:** 2012-06-22

**Authors:** Amy S Wilson, Peter P Marra, Robert C Fleischer

**Affiliations:** 1 Migratory Bird Center, Smithsonian Conservation Biology Institute, 3001 Connecticut Ave N.W, Washington, DC, 20008, USA; 2 Center for Conservation and Evolutionary Genetics, Smithsonian Conservation Biology Institute, 3001 Connecticut Ave N.W, Washington, DC, 20008, USA

## Abstract

**Background:**

Kirtland’s warblers are the rarest songbird species in North America, rarity due in part to a reliance on early successional Jack Pine forests. Habitat loss due to fire suppression led to population declines to fewer than 200 males during the 1970s. Subsequent conservation management has allowed the species to recover to over 1700 males by 2010. In this study, we directly examine the impact that low population sizes have had on genetic variation in Kirtland’s warblers. We compare the molecular variation of samples collected in Oscoda County, Michigan across three time periods: 1903–1912, 1929–1955 and 2008–2009.

**Results:**

In a hierarchical rarified sample of 20 genes and one time period, allelic richness was highest in 1903–1912 sample (a_r_ = 5.96), followed by the 1929–1955 sample (a_r_ = 5.74), and was lowest in the 2008–2009 sample (a_r_ = 5.54). Heterozygosity measures were not different between the 1929–1955 and 2008–2009 samples, but were lower in the 1903–1912 sample. Under some models, a genetic bottleneck signature was present in the 1929–1955 and 2008–2009 samples but not in the 1903–1912 sample.

**Conclusions:**

We suggest that these temporal genetic patterns are the result of the declining Kirtland’s warbler population compressing into available habitat and a consequence of existing at low numbers for several decades.

## Background

Two fundamental concepts within conservation genetics are that *i*) genetic diversity is important for population persistence and *ii*) that the amount of genetic diversity is mostly determined by the effective population size (N_e_), which is typically much smaller than the census size (N_c_) [1]. Theoretical models predict that severe reductions in population size termed as population bottlenecks, have a significant impact on the N_e_, and thus the genetic diversity of populations, so understandably, species that have undergone bottlenecks are often the focus of genetic studies. Significant bottlenecks occurred in populations of black-footed ferrets (*Mustela nigripes,* n = 18*,*[2]) whooping cranes (*Grus Americana,* n = 14, [3]), and northern elephant seal (*Mirounga angustirostris,* n = 10-30*,*[4]), and all three species have low genetic variation within the contemporary populations. However, the short-tailed albatross *Phoebastria albatrus*, which declined to 50–60 individuals, has high levels of genetic variation [5].

The genetic consequences of population bottlenecks can be directly assessed when pre-bottleneck levels of genetic variation can be measured and compared to contemporary samples. For example, temporally spaced samples were used to demonstrate that the loss of mtDNA variation in the nēnē (*Branta sandvicensis*) occurred during prehistoric times, and not during more recent declines [6]. Recent declines did however, impact genetic diversity in greater prairie chickens (*Tympanuchus cupido)*[7], but only influenced genetic structure in peregrine falcon (*Falco peregrinus*) populations [8].

**Figure 1:**
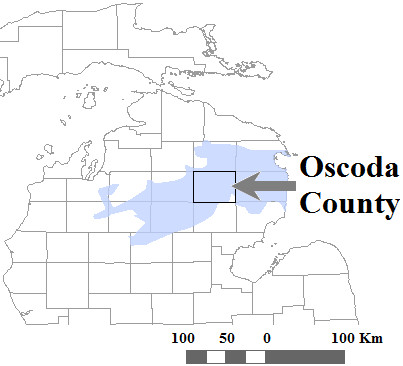
**Map of breeding distribution of Kirtland’s warbler (*Dendroica kirtlandii*) in Michigan.** The boundaries of Oscoda County which is the focal sampling locality for this study is highlighted.

**Figure 2:**
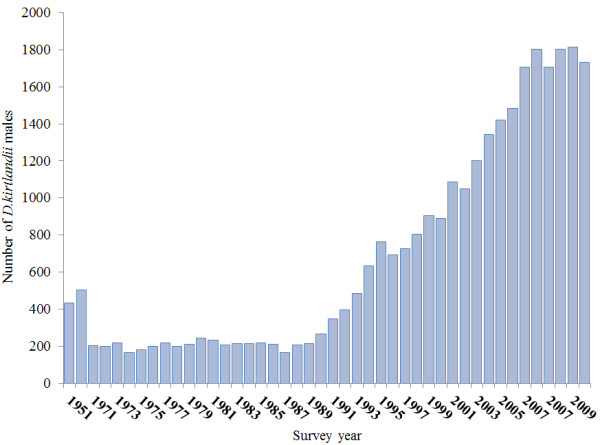
Total counts for annual census of male Kirtland’s warblers from 1951–2010 in the breeding season in Michigan.

Kirtland’s warbler (*Dendroica kirtlandii*) is the rarest songbird in North America, with a history of rarity and population declines. Kirtland’s warblers are Neotropical migrants that during breeding, specialize on early-successional stands of jack pine (*Pinus banksiana*) in the lower peninsula of Michigan, and overwinter in the Bahamian archipelago (Figure 1) [9-11]. Historical records, and estimates of past habitat availability, suggest that Kirtland’s Warblers were not a common species in the 19^th^ century, perhaps numbering less than 5000 birds [12]. However, in the 1940s, Kirtland’s Warbler populations began to decline markedly, consisting of only 530 males in the 1950s. The decline is likely because on the breeding grounds, Kirtland’s warbler abundance is closely linked to the incidence of large-scale wildfires that generate the early successional jack pine habitat on which these warblers are specialized [12]. The absence of large fires during 1946–1980 reduced the amount of early-successional jack pine stands, which, when compounded with brood parasitism by the brown-headed cowbird (*Molothrus ater*), had severe demographic consequences for Kirtland’s warblers. Kirtland’s warblers were listed as an endangered species in 1967, and by 1971, only 201 Kirtland’s warbler males were counted, which was down from the 502 males counted in 1961 (Figure 2). This alarming decline led to cowbird control measures, which likely prevented extinction, but Kirtland Warbler populations only began to increase after several large fires increased the availability of larger tracts of suitable habitat [10,13]. Fortunately, these management efforts have resulted in the Kirtland’s warbler populations increasing to an estimated 1733 males in 2010.

In this study we have two objectives, we first measure and compare the genetic diversity from samples collected in Oscoda County, Michigan (Figure 1), across three time periods: 1903–1912, 1929–1955 and 2008–2009. Secondly, we determine if the scenario inferred from the molecular data is concordant with the historical census data and if any additional insight could be gained into the population structure of the declining Kirtland’s warbler population.

## Results

### Genetic diversity

After correcting for the multiple tests, none of the loci departed from Hardy-Weinberg equilibrium, and all loci were also in linkage equilibrium. The null allele and dropout tests implemented by microchecker did not suggest that large allele drop out or null alleles were an issue. In the hp-rare hierarchical rarefaction, the rarefied sample was limited to 20 genes and one time period. The hierarchical rarefaction provides three estimates of allelic richness for three pooled time periods (2008–2009, 1929–1955 and 1903–1912) and six estimates for 3–4 year subdivided intervals. The group-level estimates of allelic richness for the three time periods were lowest in the contemporary population (2008–2009, a_r_ = 5.54), followed by the middle sampling period (1929–1955, a_r_ = 5.74) and highest in the early sampling (1903–1912, a_r_=5.96). The allelic richness estimates for the subdivided intervals was similar in the 1903–1912, 1929–1932, 1934–1938 and 1940–1945 samples, but the 1951–1955 sample had lower allelic richness than all other intervals. With the exception of 1951–1955, the 2008–2009 sample had the next lowest estimate of allelic richness (Table 1).

**Table 1:** Comparison of population-level allelic richness and individual-level genetic diversity (IR and PHt) for historical (1903–1912), (1929–1955) and contemporary (2008–2009) samples

	**Year interval**	**AR**	**PAR**	**IR**	**PHt**
**Early**	1903-1912	5.96	1.12	0.11	0.61
**Middle**	1929-1932	6.00	0.87	−0.08	0.77
	1934-1938	6.17	0.94	−0.02	0.71
	1940-1945	6.08	0.90	−0.06	0.75
	1951-1955	4.71	0.48	0.04	0.68
**Contemporary**	2008-2009	5.54	0.84	0.0	0.73

On a group-level, private allelic richness was highest in the early sample (1903–1912) (ar_P_ = 1.12), while private allelic richness in the middle (ar_P_ = 0.80), and contemporary populations (ar_P_ = 0.84), were comparable. The five subdivided estimates between 1929 and 1955 are slightly variable with the 1951–1955 sample being very low (Table 1).

**Figure 3:**
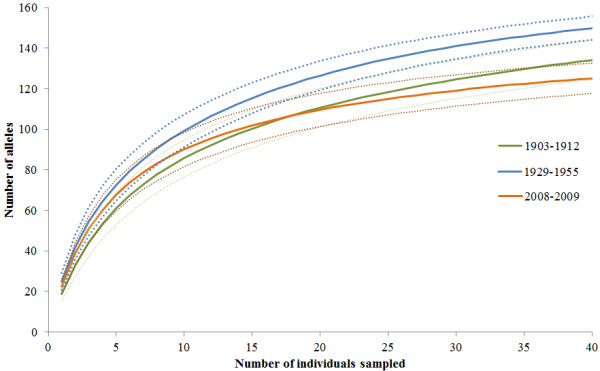
**Allelic accumulation curve for*Dendroica kirtlandii*populations in time intervals of 1903–1912, 1929–1955, and 2008–2009.** The upper and lower limits of the 95% confidence intervals are shown with a dotted line.

The allelic accumulation curve illustrates that as the rarefied sample size increased, the total number of distinct alleles within each sample became statistically different between the 2008–2009 and 1929–1955 samples at a rarefied sample of 21 individuals (Figure 3). As a comparison, at a rarefied sample size of 45, the allelic richness of the 1903–1912 population is 138 (95% CI 128.1-148), 1929–1955 population is 153 (95% CI 147–159), and the 2008–2009 sample has an allelic richness of 127 (95% CI: 120–135). So these analyses suggest that genetic variation was lost during the sampling periods of 1929–1955 and 2008–2009.

Individual heterozygosity (PHt) was not statistically different between time periods. The internal relatedness, was significantly higher within individuals in the 1903–1912 time sample (0.11 (95% CI: 0.09, 0.22)), compared to the average of the 1929–1955 samples (−0.03 (95% CI: 0.041, -0.078)) and 2008–2009 (0.0 (95% CI: - 0.03,^-^0.04)) samples (Table 1). Higher values of internal relatedness suggest that the parents of a particular individual were more closely related than another individual with a lower internal relatedness.

### Direct inference of N_e_

Based on the Kirtland’s warbler census data from 1971–2008, the effect of the population decline would have reduced the effective size of the Kirtland’s warbler population by approximately 52%, with a maximal long-term N_e_ of approximately 700. This estimate assumes that for each counted male, there was an uncounted female and assuming that no factors increasing the N_e_ are influential in the population.

### Molecular inference of N_e_

The molecular inference of N_e_ based on the linkage disequilibrium method for the 2008–2009 sample was N_e_ =161 (100–296). The N_e_ point estimate for 1929–1955 was N_e_ = 259 (128–4131), but for the 1903–1912 sample, the estimate was indeterminable likely because of missing data, or because the true N_e_ may be larger than what can be precisely estimated with this method [14]. The temporal estimates of N_e_ provided comparable estimates of the harmonic N_e_ spanning from 1903 to 2008, both when two (1903–1912, 2008–2009: N_e_ =1134 (855–1375)); and three (1903–1912, 1929–1955, 2008–2009: N_e_ =945 (786–1309)); sampling periods were used in the analysis. The temporal estimates of N_e_ range from 786 to 1375, but the lower limits of these large confidence intervals are somewhat close to the direct estimate of N_e_ ~700.

### Genetic bottleneck test

There was no significant heterozygosity excess in the 1903–1912 sample under all three mutation models (TPM, SMM and IAM). The sample from 1929–1955 only had support for significant heterozygosity excess under the IAM models for both the Wilcoxon (p = 0.019) and sign test (p = 0.009). Similarly, the sample from 2008–2009 had significant heterozygosity excess assuming an IAM mutation model (p = 0.0016) for the Wilcoxon and sign tests (p = 0.003). Under the TPM and SMM models, neither the 1929–1955 or 2008–2009 periods had significant heterozygosity excess. All time periods showed a normal L-shaped allele frequency distribution.

### Structure analyses

**Figure 4:**
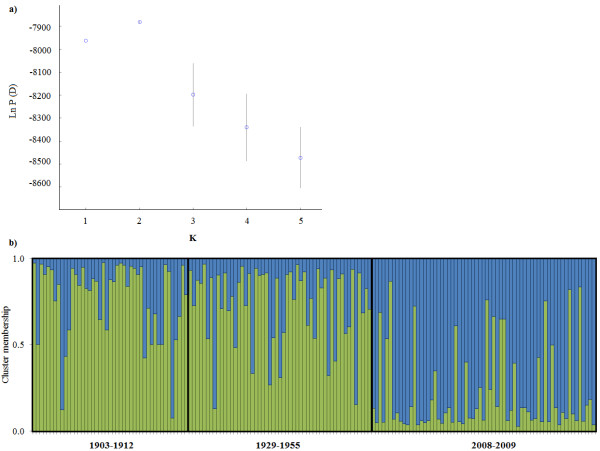
**Results of structure analyses for Kirtland’s warbler samples collected from Oscoda County, Michigan in three time intervals: 1903–1912, 1929–1955 and 2008–2009.** A model of K = 2 was most supported. Each column represents an individual where cluster membership assignment is on the y-axis.

The cluster analyses as implemented by structure identified the most probable model as K = 2 (Figure 4a). Under the K = 2 model, cluster membership distributions were similar across individuals in the 1903–1912 and 1929–1955 sampling periods. In the 2008–2009 sampling period, the cluster membership assignments shifted towards the second cluster (Figure 4b).

### Population simulations

**Figure 5:**
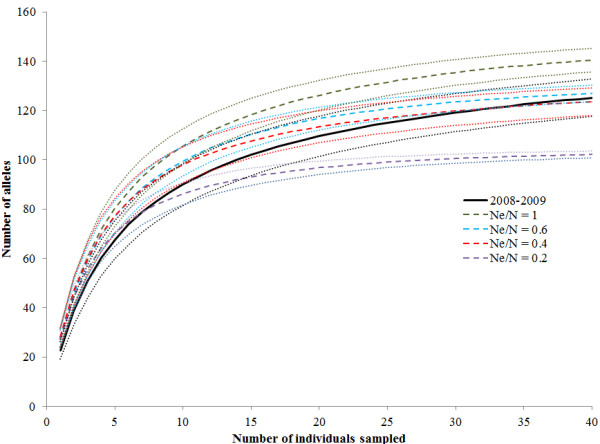
**Allelic accumulation curve for Kirtland’s warbler population sampled in 2008/2009 and four simulated populations based on N**_**e**_**/N**_**C**_**ratios of 1, 0.6, 0.4 and 0.2.** The upper and lower limits of the 95% confidence intervals are shown with a dotted line.

The population simulation that assumed a N_e_/N_C_ ratio of 0.4 best matched the allelic accumulation curves for the 2008–2009 sample (Figure 5). The estimated N_e_ from the final generation in the simulation was substantially larger at 620 (244-∞), but the 95% interval of the simulation overlaps the direct estimate of N_e_ under the assumptions of a N_e_/N_C_ of 0.4 (N_e0.4_ = 279) and the upper range of N_e_ for the 2008–2009 sample. For the bottleneck tests, under the IAM models, the simulated data of N_e_/N_C_ = 0.4 showed excess heterozygotes in all 17 loci, both when a subset of 53 individuals was used (p < 0.0001) and when the entire simulated final population was used (p < 0.0001).

## Discussion

Based on habitat availability and sighting records, Kirtland’s warblers were not a common species in the early 19^th^ century [10,15]. If historical estimates are accurate, a large population decline occurred sometime between 19^th^ century and the first census in the 1950s, when only 530 birds were counted. However, we don’t know if this decline occurred as a slow deterministic decline, or as a more rapid series of bottlenecks. It is possible that the greatest population decline occurred prior to 1902–1913, so comparing the variation between 1902–1913 and more contemporary samples reflect genetic effects of demography during those periods. However, between 1951 and 2009, the Kirtland’s warbler population underwent a 60% decline within a decade, followed by 20 years at a low (< 250 birds) population size. This population history resulted in a loss of allelic diversity in contemporary Kirtland’s warbler populations, which is made evident by the higher levels of diversity found in samples collected in 1903–1912 and 1929–1955. We estimate that 1.7 alleles/locus have been lost in the Kirtland’s warbler population over that time frame. In comparison, the Wisconsin population of greater prairie chicken which had undergone a 90% decline to approximately 2000 individuals, lost an estimated 2.2 alleles/locus [7]. Although the allelic richness in the early Kirtland’s warbler populations was higher than the contemporary sample, individual heterozygosity did not differ between time periods. The increased loss of allelic variation, compared to heterozygosity is an expected outcome in population bottleneck scenarios [16,17], and has been reported in other species [7,18].

The consequences of the Kirtland’s warbler’s demographic history can also be seen in the strong temporal clustering of samples from 1903–1955 *versus* 2008–2009 (Figure 4b), which is most likely due to the influence of genetic drift. This pattern of contemporary and historic samples forming distinct genetic clusters was also reported in Dutch populations of black grouse (*Tetrao tetrix*), which also have a history of population decline [19]. For populations with reduced N_e_, genetic drift can be a strong force that leads to both a loss of genetic variation, and genetic divergence among populations [1]. In studies where historic and contemporary genetic structure can be compared, the potential impact of genetic drift becomes more evident.

What do the genetic patterns suggest about the decline process?

In populations that have undergone documented declines, molecular data can provide information on the true severity of the bottleneck, and serve as a reminder that demographically consequential events may be difficult to detect genetically [20]. It is known from census data that Kirtland’s warbler underwent a bottleneck, and that the expected signals of excess heterozygotes and reduced N_e_[21] were present in the 1929–1955 and 2008–2009 samples, but only under certain model assumptions. The absence of a bottleneck signal in the 1903–1912 sample could suggest that either Kirtland’s warblers had not yet begun to decline, or if they were declining prior to 1903–1912, the decline occurred in a slow deterministic manner rather than in a series of intense bottlenecks.

Dispersal patterns can complicate the genetic signature of a declining population. For example in populations of Fennoscandian lesser white-fronted goose (*Anser erythropus*), changes in immigration patterns led to a temporary increase in genetic variation within a declining population [22]. We suspect that changes in distribution of Kirtland’s warblers are also being reflected in the population genetic structure, which is apparent because we sampled in a single locality. However, the Bayesian cluster analysis (Figure 4b) does not provide any evidence of admixture in the 1929–1953 sample, but a caveat being that admixture signals would only be present if populations were divergent [23]. Kirtland’s warbler census data across counties also support this scenario because as habitat became more broadly available and population sizes increased, the proportion of the Kirtland’s warbler population that is located in any one county tends to be lower (Additional files 1).

Prior to 1929–1955, the Kirtland’s warbler population may have been more clustered and fragmented, which would account for the higher internal relatedness in the 1903–1912 sample. Alternatively, the higher internal relatedness in the 1903–1912 sample could be due to these specimens being collected over a more restricted area, which is possible given that only a general collection location is provided for the 1903–1912 specimens (Additional file 2).

## Conclusions

The conservation implications of the genetic variation that has been lost in Kirtland’s warblers are difficult to assess, given that there is some uncertainty in the correlation between microsatellite and genomic variation [24], and predicting the impact of these losses on the fitness of non-model organisms is still poorly understood. There are cases of species existing at low levels of molecular variation for extended periods [25,26], but it is generally believed that genetic factors do impact the capacity of a population to recover from population declines [26,27].

The N_e_ is often an important parameter for endangered species management and policy [28]. The utility of our N_e_ estimates for evaluating the short-term genetic status of the Kirtland’s warblers depends on the accuracy and precision of our contemporary N_e_ estimates Simulations suggest that it is difficult to obtain a precise estimate of N_e_ when the true N_e_ is >400 because at those sizes of N_e_, sampling error is large compared to the strength of the drift induced shifts in heterozygosity and linkage disequilibrium [21,29]. Our direct estimate of a maximal N_e_ of 700 is approaching the parameter space where N_e_ is difficult to estimate [30]. Therefore, the large confidence limits around our N_e_ estimates, limit our ability to definitively state whether the current size of Kirtland’s warbler populations can meet conservation genetic objectives such as maintaining 90% of the initial diversity for a minimum of 100 years [31], but the lower range of our N_e_ estimates are too low for genetic variability to be retained in the long-term.

Based on our indirect estimates of contemporary N_e_ in the 100–300 range, Kirtland’s warbler populations may not be large enough to safeguard against the loss of evolutionary potential [30,32,33]. Population bottlenecks can downwardly bias N_e_ estimates [34,35], but our contemporary sample is more than 20 generations away from the lowest recorded population size, so we assume that this bias is not a major influence on our estimates [35].

The ratio between N_C_ and N_e_ has pragmatic use in conservation management, provided that the N_e_/N_C_ ratio is relatively consistent across time [21,35,36]. Among a variety of common and rare species, N_e_/N_C_ estimates range from 0.1 to 0.5 [30,37,38] so that genetic management could still be necessary at surprisingly large census sizes. Our lowest estimate of the N_e_/N_C_ ratio for the contemporary Kirtland’s warbler population was approximately 0.1, in which case, target population sizes would need to exceed 5000 individuals in order to meet the N_e_ =500 recommendation as per Franklin and Frankham (1998), or even larger population targets would be needed if the recommended N_e_ = 5000 of Lynch and Lande (1998) is adopted.

Studies attempting to estimate N_e_ in other endangered avian populations also reported wide confidence limits (*e.g.*, peregrine falcons (*Falco peregrinus*), 500 < N_e_ < 1000 [39]; yellow-eyed penguin *(Megadyptes antipodes)*, 200 < N_e_ < 1000 [40], which may be problematic in cases where greater precision is needed for management decisions. Genetic monitoring holds promise for the rapid detection of major population declines [41] however, the discrepancy between biologically significant patterns and the conditions where these patterns are genetically detectable should always be considered [18,19].

## Methods

### Sample collection

For the contemporary sample, blood samples were acquired from 68 Kirtland’s warblers in the breeding seasons of 2008–2009 from an ongoing reproductive and isotopic ecology project (S. Rockwell & P.P. Marra unpublished data) in Oscoda County in the lower peninsula of Michigan (Figure 1). All procedures involved in the capture and handling of Kirtland’s warblers were conducted under permit from the USFWS and NZP IAUCUC (#09-09).

The historic DNA samples were obtained from 98 historical specimens collected in Oscoda County, 45 that were collected during 1903–1912 and 53 that were collected during 1929–1955. All historical samples used in this study were from specimens that are curated at the University of Michigan (Additional file 2). The annual census of Kirtland’s Warblers is based on transect counts under a standardized protocol [42].

By sampling in a single geographic region, we avoided issues of spatial variation being confounded with temporal variation but instead must deal with the issue that our population may not reflect species wide diversity. However, Oscoda County is located close to the center of the species distribution, and has been continually inhabited with 25-30% of the population and as such, should be representative of what is occurring at the broader species level. The population counts within Oscoda County track the total population trend for the majority of the census period (Additional file 1). Small slices (< 1 mm^2^) of the hallux were carefully removed from specimens with a sterile blade and were stored dry in a labeled screw-top tube until DNA extraction.

### Molecular methods

For contemporary samples, the DNA source was dried red blood cell pellets or whole blood stored in Queen’s lysis buffer [43]. DNA was extracted from blood samples using Qiagen DNA Easy Biosprint kits according to manufacturer’s instructions. The DNA from historic samples was extracted and stored in a dedicated ancient DNA laboratory at the Center for Conservation and Evolutionary Genetics. For historical samples, DNA was obtained from toe pad tissue using Qiagen Micro kits (Qiagen, California, USA), which we processed according to manufacturer’s instructions with the exception that 40 ng of Dithiothreitol (DTT) was added to the ATL lysis buffer.

We used 17 microsatellite loci (Table 2), 12 of these loci were developed specifically for Kirtland’s warblers [44]. Five other loci Dpu16 [45], Lswu07 [46] and Vecr04, Vecr08, Vecr10 were developed for other species [47]. We specifically chose microsatellite loci in the smaller size range to increase the probability that all loci would amplify in the varying quality of DNA. Microsatellite genotyping of contemporary samples were completed in 10 μl volumes containing 1X Promega polymerase buffer (Roche Inc.), 0.5 μM fluorescently labeled forward primer, 0.5 μM unlabeled reverse primer, 2 μM each dNTP, 1.5 mM MgCl_2_, and 0.5 U Promega GoTaq. PCR profiles were initiated with 3 min at 95°C followed 35 cycles of 30 s at 95°C, 30 s at locus-specific annealing temperatures and 45 s at 72°C and a final 15 minute extension at 72°C.

**Table 2:** **Characteristics of 17 Kirtland’s warbler (*Dendroica kirtlandii*) microsatellite DNA loci: locus designation, number of alleles observed (N**_**A**_**) and average observed (Ho) and expected heterozygosities (He)**

		1903-1912		1929-1953		2008/2009	
Locus	N_A_	**Ho**	**He**	**Ho**	**He**	**Ho**	**He**
B3	4	0.50	0.63	0.57	0.55	0.42	0.52
B12	10	0.39	0.46	0.55	0.56	0.66	0.68
B102	10	0.50	0.82	0.80	0.78	0.89	0.78
B106	11	0.78	0.83	0.96	0.82	0.88	0.77
B119	9	0.69	0.75	0.83	0.76	0.85	0.75
B124	5	0.69	0.61	0.41	0.48	0.54	0.49
C105	7	0.77	0.74	0.69	0.71	0.65	0.65
D12	11	0.91	0.83	0.86	0.83	0.84	0.84
D104	15	0.80	0.85	0.89	0.89	0.90	0.89
D109	17	0.81	0.90	0.90	0.91	0.94	0.87
D126	14	0.89	0.87	0.96	0.89	0.85	0.83
D128	15	0.87	0.82	0.87	0.85	0.90	0.81
Dpu16	13	0.76	0.84	0.86	0.82	0.71	0.75
Lswu07	6	0.74	0.77	0.78	0.74	0.54	0.74
Vecr04	4	0.37	0.38	0.92	0.60	0.56	0.43
Vecr08	7	0.12	0.11	0.21	0.20	0.46	0.41
Vecr10	5	0.55	0.60	0.61	0.53	0.58	0.55

All historic PCR reactions were prepared and sealed in the dedicated ancient DNA laboratory, and transferred to the modern genetic lab for PCR thermocycling. Microsatellite genotyping of museum specimens were performed in 25 μl volumes containing 1X AmpliTaq Gold DNA polymerase buffer (Applied Biosystems, Inc.), 25 μM fluorescently labeled forward primer, 25 μM unlabeled reverse primer, 2 μM each dNTP, 10 mM MgCl_2_, and 0.5 U AmpliTaq Gold polymerase. PCRs were performed on DNA Engine Tetrad® 2 (BioRad) using a PCR profile that started with an initial 6 min at 95°C followed 50 cycles of a 45 s at 95°C, 45 s at 50°C (for all loci), 45 s at 72°C and a final 15 minute extension at 72°C.

Both contemporary and historic reaction sets included a PCR negative control and an extraction blank. All historical samples were repeated in duplicate, with one locus B3 that was repeated in triplicate. There were three individuals where a third ghost allele appeared in one replicate and these individuals were repeated a third time. Across all individuals and all loci, 10% of data was missing or edited, in each time period. Amplification products were analyzed in an ABI 3100 automated DNA sequencer (Applied Biosystems, Inc.) using GeneScan® 3.7 (Applied Biosystems, Inc.). Fragment sizes were sized using Diamond ROX 500 bp size standards (Applied Biosystems, Inc.) and scored in Genemapper® Software v 4.1 (Applied Biosystems, Inc.)

### Analytical methods

We tested for null alleles and dropout using the program microchecker[48]. This was particularly important for the historical samples because their lower DNA concentrations and fragmented DNA increases the likelihood of allelic drop-out particularly for large alleles. We tested for linkage disequilibrium and Hardy-Weinberg equilibrium using the program genepop 007 [49] and used the false discovery rate [50], to correct for multiple comparisons.

### Genetic diversity analyses

Estimates of allelic richness are heavily dependent on sample size and so rarefaction methods are necessary. We used the rarefaction program hp-rare to estimate allelic richness and private allelic richness (alleles unique to a particular sample) in each of the three time periods [51]. hp-rare enables users to conduct hierarchical rarefaction so we further subdivided the time samples into six groups: 1903–1912, 1929–1932, 1934–1938, 1940–1945, 1951–1955 and 2008–2009. This subdivision enabled us to control for the larger number of time periods sampled the time period of 1929–1955 (referred to as ‘middle’). As a complement we also used the R package ares[52] which is rarefaction program that also calculates the 95% confidence limits to the allelic richness estimates. We used the 95% confidence limits to determine if differences between groups exceed the variation due to sampling error within groups. We used the program genhet[53] to calculate the proportion of heterozygous loci (PHt) and the internal relatedness (an estimate of parental relatedness) [54]. Statistical significance of differences among time samples was determined using general linear models in Program R 2.13.0 [55] Population level estimates of heterozygosity were obtained from gda v 1.0 [14].

### Estimation of effective population sizes

#### Direct inference of N_e_

We estimated the size of the contemporary N_e_ for Kirtland’s warblers using direct demographic methods as well as ones that infer N_e_ from molecular data. The Kirtland’s warbler census data only estimates the number of singing males, so the number of females and total population size in each year is unknown. Passeriformes tend to have male-biased sex ratios [56], so we calculated estimates of N_e_ based on a 1:1 ratio or a 1:2 male-biased ratio. Our estimate of N_e_ will be an overestimate as we are not incorporating other demographic factors (*i.e.* reproductive variance, sex ratios, age-class distributions) that further affect N_e_[21,57], We estimated the relative impact that the fluctuations in population size during 1971–2010 had on the overall N_e._ The effect of population fluctuations on N_e_ is the quotient of the harmonic mean *versus* the arithmetic mean size, such that the smallest N_e_ will have disproportionately large effects on the cumulative N_e_[36].

#### Molecular inference of N_e_

We used the data from 17 microsatellites to estimate the contemporary N_e_ and historical N_e_. To infer the contemporary N_e_ in each time period, we used the linkage disequilibrium method (LD) as implemented in the program LDN_e_, [58]. The LD method is based on the theoretical expectation that when N_e_ decreases, linkage disequilibrium due to drift will increase [59,60]. In the LDN_e_ analysis, we only included alleles exceeding a frequency of 0.02. We also used temporal alleles methods to estimate N_e_, with the moments based approach [60]. These analyses assume that N_e_ is stable during the sample period, which may not hold for our historic samples that were collected over a nine and 25-year period respectively. Analyses were run using the program N_e_estimator[61].

#### Genetic bottleneck test

We tested for a signal of a genetic bottleneck in each of the three time periods using the program bottleneck[62]. The tests in program bottleneck are based on the expectation that a population bottleneck will lead to a rapid loss of rare alleles producing an excess of heterozygotes and a shift in allele frequency proportions [63]. These expectations were tested under all three available mutation models: the infinite alleles model (IAM), the stepwise mutation model (SMM) and the two-phased model (TPM) of mutation, the latter of which is thought to best fit microsatellite data [62] and recent bottleneck events [64]. We ran 1000 replications and used a TPM composed of 95% SMM and 5% IAM and a variance of 12 as suggested by the program authors [62]. The significance of any deviations from mutation-drift equilibrium was based on the Wilcoxon signed-rank test and a standardized differences test. We also used the mode-shift test as implemented in bottleneck. The mode-shift test determines if the allele frequency distribution has been shifted towards more common alleles with fewer low frequency alleles as would be expected in the case of a bottleneck.

#### Structure analyses

Because we suspected that population compression during the 1929–1955 time period may have influenced Kirtland’s warbler genetic structure, we also ran a cluster analysis as implemented in the program Structure 2.3.3 [[23]], to determine if population genetic structure varied between the sampled time intervals. We ran structure for 10 replicates across K = 1 to K = 5 with each run consisting of an initial burn-in of 1 x 10^5^ iterations, with 1 x 10^6^ iterations under the correlated allele frequency model and with an uninformative prior on the temporal sampling period. The program structure harvester v0.3 was used to process the structure results files [65] and clummp v1.2.2 [[66]] was used to summarize across the replicate runs for the most probable K value. The most supported value of K was inferred from the posterior probabilities [67] and the ΔK method [68].

#### Population simulations

Using actual census data we simulated the Kirtland’s warbler population in bottlesim v2.6 [69] from 1971 to 2008, and compared the final year from this simulation to our data from 2008–2009. Simulations were initiated with the allelic frequencies found in the 1929–1955 sample, an average life expectancy of four years and a generation time of one year [9], 50% overlap in generation overlap and random mating. Due to a lack of the data necessary for generation time estimation for Kirtland’s Warblers, we used the age of maturity as a proxy for generation time.

We ran four simulations based on the Kirtland’s warbler male census data and assuming a 1:1 sex ratio with N_e_/N_C_ ratios of 1, 0.6, 0.4 and 0.2. Although the accuracy of the census data has been questioned, it is at least accepted as a relative measure of abundance [70]. Each simulation consisted of 1000 iterations across the 58 generations and output was a simulated set of genotypes for the entire population in the final simulated year. We then took a random subsample of 53 individuals from the simulated genotypic data and analyzed the allelic richness in the subsample using ares[50], N_e_estimator[61] and bottleneck[62].

## Competing interests

The authors declare that they have no competing interests.

## Authors' contributions

ASW carried out the molecular genetic work and analyses and drafted the manuscript. RCF participated in molecular genetic work, genetic analyses and manuscript writing. PM conceived the study, participated in its data collection and helped to complete the manuscript. All authors read, provided substantial edits and approved the final manuscript.

## Supplementary Material

Additional file 1 **Appendix 1.** Proportion of total male population of Kirtland’s warblers located in each county in 1951, 1981 and 2005. figure depicting the distribution of Kirtland’s warblers based on annual survey data. Click here for file

Additional file 2 **Appendix 2.** Sampling localities and collection dates of Kirtland’s warblers sampled from Michigan State University. Click here for file
