# Supplementary material for: Temporal patterns of genetic diversity in Kirtland’s warblers (Dendroica kirtlandii), the rarest songbird in North America
Source: BMC Ecol. 2012 Jun 22;12:8. doi: 10.1186/1472-6785-12-8 (PMC3430571; doi:10.1186/1472-6785-12-8)

**Appendix 1.**

# Proportion of the total male population of Kirtland’s warblers located in each county in Michigan state during 1951, 1981 and 2005. Oscoda County is indicated with an asterisk.


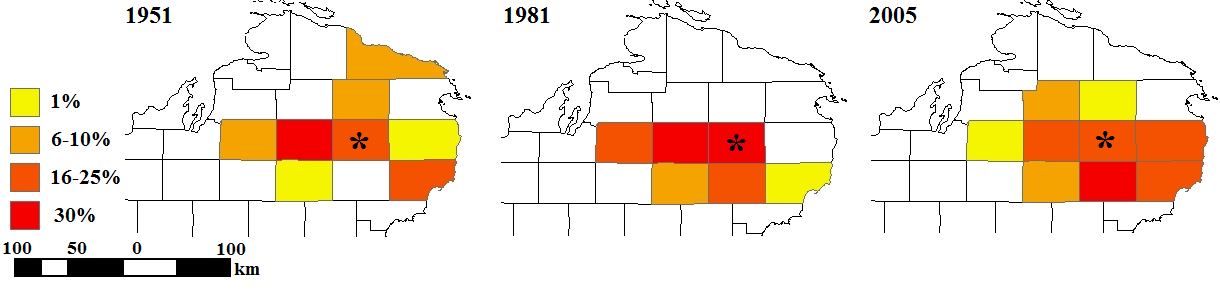

Supplement: Additional file 1 — Appendix 1. Proportion of total male population of Kirtland’s warblers located in each county in 1951, 1981 and 2005. figure depicting the distribution of Kirtland’s warblers based on annual survey data. [file 1472-6785-12-8-S1.docx]
