# Supplementary material for: Temporal patterns of genetic diversity in Kirtland’s warblers (Dendroica kirtlandii), the rarest songbird in North America
Source: BMC Ecol. 2012 Jun 22;12:8. doi: 10.1186/1472-6785-12-8 (PMC3430571; doi:10.1186/1472-6785-12-8)
Supplement: Additional file 2 — Appendix 2. Sampling localities and collection dates of Kirtland’s warblers sampled from Michigan State University. [file 1472-6785-12-8-S2.docx]

**Appendix 2:** Sampling localities and collection dates of Kirtland’s Warblers sampled from Michigan State University.

| Catalog Number | Collection date | Collection locality | Collector name |
| --- | --- | --- | --- |
| 30966 | 2/7/1903 | Parmalee, Oscoda County | Wood, Norman A |
| 31284 | 11/7/1903 | no specific locality, Oscoda County | Wood, Norman A |
| 31287 | 11/7/1903 | no specific locality, Oscoda County | Wood, Norman A |
| 125676 | 7/14/1909 | Luzerne, Oscoda County | Peet, Max Minor |
| 125677 | 7/14/1909 | Luzerne, Oscoda County | Peet, Max Minor |
| 125678 | 7/15/1909 | Luzerne, Oscoda County | Peet, Max Minor |
| 125679 | 8/11/1909 | Luzerne, Oscoda County | Peet, Max Minor |
| 208756 | 7/9/1909 | Mio, Oscoda County | Peet, Max Minor |
| 208757 | 7/11/1909 | Mio, Oscoda County | Peet, Max Minor |
| 208758 | 7/14/1909 | Mio, Oscoda County | Peet, Max Minor |
| 125681 | 7/5/1910 | no specific locality, Oscoda County | Peet, Max Minor |
| 125684 | 7/10/1910 | Luzerne, Oscoda County | Peet, Max Minor |
| 125685 | 7/10/1910 | Luzerne, Oscoda County | Peet, Max Minor |
| 125686 | 7/10/1910 | Luzerne, Oscoda County | Peet, Max Minor |
| 125687 | 7/10/1910 | Luzerne, Oscoda County | Peet, Max Minor |
| 125688 | 7/11/1910 | Luzerne, Oscoda County | Peet, Max Minor |
| 125689 | 7/12/1910 | Luzerne, Oscoda County | Peet, Max Minor |
| 125690 | 8/1/1910 | Luzerne, Oscoda County | Peet, Max Minor |
| 125691 | 8/8/1910 | Luzerne, Oscoda County | Peet, Max Minor |
| 125692 | 8/9/1910 | Luzerne, Oscoda County | Peet, Max Minor |
| 125693 | 8/9/1910 | Luzerne, Oscoda County | Peet, Max Minor |
| 125694 | 8/10/1910 | Luzerne, Oscoda County | Peet, Max Minor |
| 125695 | 8/10/1910 | Luzerne, Oscoda County | Peet, Max Minor |
| 125696 | 8/10/1910 | Luzerne, Oscoda County | Peet, Max Minor |
| 125697 | 8/10/1910 | Luzerne, Oscoda County | Peet, Max Minor |
| 125698 | 8/11/1910 | Luzerne, Oscoda County | Peet, Max Minor |
| 125699 | 8/11/1910 | Luzerne, Oscoda County | Peet, Max Minor |
| 125700 | 8/11/1910 | Luzerne, Oscoda County | Peet, Max Minor |
| 125702 | 8/12/1910 | Luzerne, Oscoda County | Peet, Max Minor |
| 208759 | 8/12/1910 | Luzerne, Oscoda County | Peet, Max Minor |
| 125703 | 8/26/1911 | Luzerne, Oscoda County | Peet, Max Minor |
| 125704 | 8/31/1911 | Luzerne, Oscoda County | Peet, Max Minor |
| 125705 | 8/29/1912 | Luzerne, Oscoda County | Peet, Max Minor |
| 125706 | 8/29/1912 | Luzerne, Oscoda County | Peet, Max Minor |
| 125707 | 8/29/1912 | Luzerne, Oscoda County | Peet, Max Minor |
| 125708 | 8/29/1912 | Luzerne, Oscoda County | Peet, Max Minor |
| 125709 | 8/30/1912 | Luzerne, Oscoda County | Peet, Max Minor |
| 125710 | 9/3/1912 | Luzerne, Oscoda County | Peet, Max Minor |
| 125711 | 9/4/1912 | Luzerne, Oscoda County | Peet, Max Minor |
| 125712 | 9/5/1912 | Luzerne, Oscoda County | Peet, Max Minor |
| 125713 | 9/5/1912 | Luzerne, Oscoda County | Peet, Max Minor |
| 125714 | 9/6/1912 | Luzerne, Oscoda County | Peet, Max Minor |
| 125715 | 9/7/1912 | Luzerne, Oscoda County | Peet, Max Minor |
| 125716 | 9/7/1912 | Luzerne, Oscoda County | Peet, Max Minor |
| 125717 | 9/8/1912 | Luzerne, Oscoda County | Peet, Max Minor |
| 71146 | 5/20/1929 | Red Oak, Oscoda County | Van Tyne, Josselyn |
| 97791 | 8/19/1929 | Red Oak, Oscoda County | Tinker, A.D. |
| 152174 | 5/20/1929 | Red Oak, 1 mi W, Oscoda County | Van Tyne, Josselyn |
| 75062 | 7/28/1930 | Red Oak, 1 mi W, Oscoda County | Sutton, George M |
| 75063 | 7/27/1930 | Red Oak, 1 mi W, Oscoda County | Sutton, George M |
| 230221 | 6/21/1931 | Luzerne, 4 mi S, Oscoda County | RupChand, Thakur |
| 230222 | 6/21/1931 | Red Oak, Oscoda County | RupChand, Thakur |
| 230223 | 6/21/1931 | Red Oak, Oscoda County | RupChand, Thakur |
| 90284 | 5/28/1932 | Red Oak, 1 mi W, Oscoda County | Brodkorb, Pierce |
| 90285 | 5/28/1932 | Red Oak, 1 mi W, Oscoda County | Brodkorb, Pierce |
| 90286 | 5/28/1932 | Red Oak, 1 mi W, Oscoda County | Brodkorb, Pierce |
| 93973 | 5/26/1934 | Red Oak, Oscoda County | Van Tyne, Josselyn |
| 96394 | 6/11/1934 | Red Oak, 4 mi NE, Oscoda County | Morrill, Ralph E |
| 104845 | 7/22/1935 | Greenwood Twp, T28N, R1E, Sec 1, Oscoda County | Van Tyne, Josselyn |
| 104982 | 6/21/1936 | T28N, R2E, S5, Oscoda County | Van Tyne, Josselyn |
| 108693 | 6/16/1937 | T27N, R2E, S6, SW part, Oscoda County | Van Tyne, Josselyn |
| 108694 | 6/14/1937 | Mio, 8 mi SE, T25N, R3E, S12, Oscoda County | Van Tyne, Josselyn |
| 108695 | 6/14/1937 | Mio, 8 mi SE, T25N, R3E, S12, Oscoda County | Van Tyne, Josselyn |
| 108696 | 6/14/1937 | Mio, 8 mi SE, T25N, R3E, S12, Oscoda County | Van Tyne, Josselyn |
| 108697 | 6/20/1937 | Mio, 8 Mi SE, T25N, R3E, S13, Oscoda County | Van Tyne, Josselyn |
| 108698 | 6/21/1937 | Red Oak, Oscoda County | Van Tyne, Josselyn |
| 108699 | 6/21/1937 | Red Oak, Oscoda County | Van Tyne, Josselyn |
| 108700 | 6/21/1937 | Red Oak, Oscoda County | Van Tyne, Josselyn |
| 110849 | 6/13/1938 | Mio, 8 mi SE, T25N, R3E, S12, Oscoda County | Van Tyne, Josselyn |
| 110850 | 6/13/1938 | Mio, 8 mi SE, T25N, R3E, S12, Oscoda County | Van Tyne, Josselyn |
| 112818 | 6/15/1940 | Mio, 10 mi SE, T25N, R4E, S18, Oscoda County | Van Tyne, Josselyn |
| 112819 | 6/23/1940 | Mio, 8 mi SE, Oscoda County | Van Tyne, Josselyn |
| 112820 | 6/25/1940 | Mio, 8 mi SE, Oscoda County | Van Tyne, Josselyn |
| 112821 | 6/23/1940 | Mio, 8 mi SE, Oscoda County | Van Tyne, Josselyn |
| 112822 | 6/27/1940 | Mio, 9.5 mi NW, Oscoda County | Van Tyne, Josselyn |
| 112823 | 6/27/1940 | Mio, 9.5 Mi NW, T27N, R1E, S14, Oscoda County | Van Tyne, Josselyn |
| 112824 | 6/27/1940 | Mio, 9.5 Mi NW, T27N, R1E, S14, Oscoda County | Van Tyne, Josselyn |
| 113901 | 5/30/1941 | Mio, 9.5 Mi NW, T27N, R1E, S14, Oscoda County | Van Tyne, Josselyn |
| 113902 | 6/1/1941 | Mio, 9.5 Mi NW, T27N, R1E, S11, Oscoda County | Van Tyne, Josselyn |
| 113903 | 6/15/1941 | Mio, 10 Mi SE, T27N, R4E, S18, Oscoda County | Van Tyne, Josselyn |
| 114209 | 6/25/1942 | Mio, 8 Mi SE, T25N, R3E, S13, Oscoda County | Van Tyne, Josselyn |
| 114210 | 6/25/1942 | Mio, 6.5 mi SE, T25N, R3E, S16, Oscoda County | Van Tyne, Josselyn |
| 114211 | 6/19/1942 | Mio, 8 mi SE, T25N, R3E, S12, Oscoda County | Van Tyne, Josselyn |
| 114628 | 6/23/1943 | Red Oak, 4 mi NE, Oscoda County | Van Tyne, Josselyn |
| 114629 | 6/26/1943 | Mio, 6 mi S, Oscoda County | Van Tyne, Josselyn |
| 114630 | 6/23/1943 | Red Oak, 4 mi NE, Oscoda County | Van Tyne, Josselyn |
| 114631 | 6/26/1943 | Mio, 6 mi S, Oscoda County | Van Tyne, Josselyn |
| 116273 | 6/18/1944 | Mio, 8 mi SE, S14, NE Corner, Oscoda County | Van Tyne, Josselyn |
| 116275 | 6/30/1944 | Mio, 8 mi SE, T25N, R3E, S14, Oscoda County | Van Tyne, Josselyn |
| 119806 | 6/15/1945 | Mio, 7 Mi SSE, T25N, R3E, S15, NE Oscoda County | Van Tyne, Josselyn |
| 119807 | 6/12/1945 | Red Oak, NE of, Sec 14, Oscoda County | Van Tyne, Josselyn |
| 136640 | 6/25/1951 | Mack Lake, W end, Oscoda County | Van Tyne, Josselyn |
| 151002 | 5/12/1952 | Mio, 6 mi S, Oscoda County | Van Tyne, Josselyn |
| 151003 | 5/12/1952 | Mio, 6 mi S, Oscoda County | Van Tyne, Josselyn |
| 151594 | 8/30/1952 | Mio, 5 Mi SSE, Oscoda County | Berger, Andrew J |
| 151595 | 8/4/1952 | Mio, 5 Mi SSE, Oscoda County | Van Tyne, Josselyn |
| 153877 | 1/11/1955 | T25N, R3E, S8, Oscoda County | Berger, Andrew J |
| 154666 | 10/15/1955 | T25N, R3E, S8, Oscoda County | Berger, Andrew J |
